# Shape Reconstruction Based on a New Blurring Model at the Micro/Nanometer Scale

**DOI:** 10.3390/s16030302

**Published:** 2016-02-27

**Authors:** Yangjie Wei, Chengdong Wu, Wenxue Wang

**Affiliations:** 1 College of Computer Science and Engineering, Northeastern University, Shenyang 110819, China; 2 State Key Laboratory of Robotics, Shenyang Institute of Automation, Chinese Academy of Science, Shenyang 110014, China; wangwenxue@sia.cn; 3 College of Information Science and Engineering, Northeastern University, Shenyang 110819, China; wuchengdong@ise.neu.edu.cn

**Keywords:** blurred imaging model, 3D shape, heat diffusion, micro/nanometer scale reconstruction

## Abstract

Real-time observation of three-dimensional (3D) information has great significance in nanotechnology. However, normal nanometer scale observation techniques, including transmission electron microscopy (TEM), and scanning probe microscopy (SPM), have some problems to obtain 3D information because they lack non-destructive, intuitive, and fast imaging ability under normal conditions, and optical methods have not widely used in micro/nanometer shape reconstruction due to the practical requirements and the imaging limitations in micro/nano manipulation. In this paper, a high resolution shape reconstruction method based on a new optical blurring model is proposed. Firstly, the heat diffusion physics equation is analyzed and the optical diffraction model is modified to directly explain the basic principles of image blurring resulting from depth variation. Secondly, a blurring imaging model is proposed based on curve fitting of a 4th order polynomial curve. The heat diffusion equations combined with the blurring imaging are introduced, and their solution is transformed into a dynamic optimization problem. Finally, the experiments with a standard nanogrid, an atomic force microscopy (AFM) cantilever and a microlens have been conducted. The experiments prove that the proposed method can reconstruct 3D shapes at the micro/nanometer scale, and the minimal reconstruction error is 3 nm.

## 1. Introduction

Nowadays, micro/nanometer scale observation, as an enabling technology in nanotechnology, is important for researchers to understand the shapes, characteristics, and interactions between two objects during micro/nano manipulation [1,2,3,4]. Therefore, observation techniques at the micro/nanometer scale, especially three-dimensional (3D) and non-destructive techniques, have great significance to explore new manipulation methods, to develop new system testing techniques, and to model dynamics and kinematics [5].

The normal tools used in micro/nanometer observation include transmission electron microscopy (TEM), scanning electron microscopy (SEM), scanning probe microscopy (SPM), scanning tunneling microscopy (STM), and optical microscopy. SEM, as an electron microscope, uses a focused beam of electrons to scan a sample, and the resolution of its images is less than 1 nm. However, it has to work under vacuum conditions. Besides, the size of its samples must fit in the specimen chamber; TEM is capable of 3D imaging and can observe the objects with sub-nanometer resolution. It is necessary to carry out scanning imaging, which is characterized by high adaptability and low cost. However, sample preparation for a TEM system is a complex procedure, because the thickness of the specimens has to be on the order of hundreds of nanometers; SPM is a microscopy technique that produces images of surfaces using a physical probe to scan the sample. The samples scanned by SPM could be in air at standard temperature and pressure or submerged in a liquid reaction environment. However, due to the scanning process, the scanning speed of SPM is generally slower in acquiring images, as is STM. Compared to them, the requirements of optical microscopes, including manipulation environment and equipment cost, are all lower than those of the previous microscopes [6]. Besides, benefitting from their real-time and non-destructive imaging ability, it is possible to use optical microscopes to achieve real-time vision feedback and to improve manipulation precision in micro/nano technology [7,8].

However, most recent optical microscopes cannot obtain a 3D image directly, and the 3D reconstruction methods based on optical microscopes at the macro scale, including depth from focus (DFF), depth from stereo (DFS), and depth from defocus (DFD), have some problems in reconstruction at the micro/nanometer scale [9]. For example: (1) the manipulation space in a nanomanipulation is too limited to set up two optical microscopes for observing an object at the same time, while DFS and the traditional DFD estimate depth information use images obtained with two or more cameras at different positions, with different orientations or with different camera parameters, of the same scene [10,11,12]; (2) For an optical microscope, the depth of field is inversely proportional to its resolution. Therefore, the depth of field of a high resolution microscopy is very short, and it is difficult to capture a sequence of images with different depth information, as required by DFF. Besides, to capture a sequence of images is time-consuming, therefore, DFF is difficult to use in real-time applications [13]; (3) The imaging property of a high resolution microscopy is too complicated to be described by the principles of light-traveling at the macro scale, and the traditional geometrical optics-based techniques have not considered the distribution property of intensity in high resolution microscopy. However, the intensity distribution is the theoretical base of optical imaging. Lacking an understanding of the intensity distribution, it is difficult to describe the imaging process and to improve the resolution of reconstruction theoretically. Therefore, in order to reconstruct the 3D shape of an object at the micro/nanometer scale, a method that can relate intensity distribution of a source point and depth variation is needed in real applications.

In response to these issues, a shape reconstruction method based on optical techniques at the micro/nanometer scale is proposed in this paper. Our method provides a mathematical model between optical intensity distribution and depth information, and our contribution can be described as follows: first, the heat diffusion physics equation is analyzed and an optical diffraction model is modified to explain the basic principles of the blurred imaging process resulting from depth variation. Second, a new blurring imaging model is achieved based on curve fitting of a 4th order polynomial curve. The heat diffusion equations are introduced to solve the process of imaging blurring, taking into account global optimization. Finally, a series of experiments are conducted and the results prove that our proposed method can reconstruct 3D shapes at the micro/nanometer scale.

## 2. Heat Diffusion and Blurring Imaging

### 2.1. Heat Diffusion in Physics

In physics, heat diffusion of most fluids and some homogeneous solid materials like gels is the same in every direction, and it is called isotropic heat diffusion, characterized by a single diffusion coefficient *ε*.

First, assume that the concentration is *u* and flux is *J*, according to Fick’s first law, the relationship between the flux and the concentration gradient can be denoted as:

(1) J(x,y,t)=ε∇u(x,y,t)

where *y* and *z* are the horizontal and the vertical coordinates of a diffusion source, respectively; the intensity of diffusion is controlled by the diffusion coefficient *ε*, and it is nonnegative; *t* is the diffusion time which expends on the process of diffusion caloric; “∇” denotes the gradient operator:

(2) $$\nabla ={\left[\frac{\partial }{\partial x}\;\frac{\partial }{\partial y}\right]}^{T}$$

Then, the continuity equation, which relates the time derivative of the concentration to the divergence of the flux, can be formulated as:

(3) $$\frac{\partial u(x,y,t)}{\partial t}=\nabla \cdot J(x,y,t)$$

where “∇⋅” is the divergence operator, $\frac{\partial }{\partial x}+\frac{\partial }{\partial y}$.

Putting Equations (1) and (3) together, the diffusion equation is denoted as:

(4) $$\frac{\partial u(x,y,t)}{\partial t}=\varepsilon \nabla^{2}u(x,y,t)$$

Therefore, the isotropic heat diffusion model can be denoted as:

(5) $$\left\{ \begin{array}{l} \frac{\partial u(x,y,t)}{\partial t}=\varepsilon \nabla^{2}u(x,y,t)\\ u(x,y,0)=u_{0}(x,y) \end{array}\right.$$

where *u*_0_(*y*, *z*) is the initial condition of diffusion.

If the diffusion coefficient varies spatially, the isotropic heat diffusion is transformed into the inhomogeneous heat diffusion, and its model is obtained yielding the following equation:

(6) $$\left\{ \begin{array}{l} \frac{\partial u(x,y,t)}{\partial t}=\nabla \cdot (\varepsilon (x,y)\nabla u(x,y,t))\\ u(x,y,0)=u_{0}(x,y) \end{array}\right.$$

The inhomogeneous heat diffusion first appears in physics, and the diffusion intensity of every point in space is controlled by the heat diffusion coefficient. Therefore, as inhomogeneous diffusion activity increases, the diffusion region whose density contrast is lower will become much smoother, while the one whose density contrast is higher will not have a large change. Benefitting from this property, the heat diffusion equation has recently been used in image processing.

### 2.2. Blurring Imaging Model

On geometrical optics, there is an assumption that optical light travels in straight lines, and image blurring results from the variation of camera parameters, such as the focal length. However, in most optical systems, the imaging beam only can travel through a round hole restricted by a diaphragm. If a small object is required to be imaged clearly, we need an optical system of high magnification in theory. When the size of the object, the size of some elements in the optical system, and the wave length of imaging are in the same order of magnitude, optical light is traveling around the hole obviously. That means that the direction of the optical light is changed, and the intensity distribution of an image point is a round light spot, rather than a point as expected [14,15,16]. This phenomenon is known as optical diffraction.

**Figure 1:**
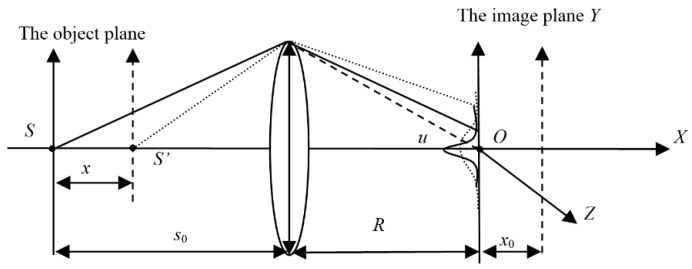
The diagrammatic sketch of the optical light traveling in an optical system.

Optical diffraction that appears in a normal optical imaging system is called Fresnel diffraction, and a diagrammatic sketch of the optical path in the optical system is shown in Figure 1, where *OXYZ* is the coordinate system; *X* is the optical axis; *O* is the origin point of the coordinate system, and *YZ* is the imaging plane. As stated in [17], if we assume *P* is a random point on the imaging plane, its amplitude can be described by an equation:

(7) $${\tilde{E}}_{P}=\frac{A}{x_{0}}\exp\left[ik(x_{0}+\frac{\rho^{2}}{2b})\right]\cdot {\sum_{n=1}^{\infty }\left(-i\frac{x_{0}}{R}\frac{a}{\rho }\right)}^{n}J_{n}(\frac{2\pi }{\lambda }\frac{a}{b}\rho )$$

where *A* the unit amplitude of *P*, *d* is the radius of lens, *ρ* is the distance between *P* and *X* axis; *J_n_* is a Bessel function of *n* order; *λ* is the wavelength of the incident light in the imaging system; *k* = 2*π*/*λ*; *R* is the distance between the lens and the imaging plane; *x* is movement of the object plane from the ideal object place along *X* axis; *x*_0_ is movement of the image plane from the ideal image place along *X* axis.

The axial magnification of the camera *m* is related to the object distance and the imaging distance, therefore *x* *=* *mx*_0_, and we modify the description in [17] with the movement of the object plane *x*:

(8) $${\tilde{E}}_{P}=\frac{A}{x}\exp\left[ik(x+\frac{\rho^{2}}{2R})\right]\cdot {\sum_{n=1}^{\infty }\left(-i\frac{x}{R}\frac{d}{\rho }\right)}^{n}J_{n}(\frac{2\pi }{\lambda }\frac{d}{R}\rho )$$

Based on the pinhole image analysis, the imaging property on the *XY* plane and the *XZ* plane are almost same. Due to the property of symmetry, parameter *ρ* in Equation (8) can be replaced by parameter *y*. Therefore, we can reduce Equations (8) to (9) and start to research the intensity distribution of a source point along the *Y* axis:

(9) $${\tilde{E}}_{P}=\exp\left[ik(x+\frac{y^{2}}{2R})\right]\cdot \left\{-i\frac{J_{1}(2\pi \lambda^{-1}y\sin u)}{\pi \lambda^{-1}y\sin u}+\frac{\lambda }{\pi \sin^{2}u}\cdot \sum_{n=2}^{\infty }J_{n}(2\pi \lambda^{-1}y\sin u){\left(-iy^{-1}\sin u\right)}^{n}x^{n-1}\right\}$$

where sinu=dR; *y* is the distance between *P* and *Y* axis.

Then, the normalized intensity distribution of a random point *P* can be denoted as:

(10) $$I_{P}={\tilde{E}}_{P}\cdot {\tilde{E}}_{P}^{*}=B^{2}$$

where B=(−iJ1(2πλ−1ysinu)πλ−1ysinu+λπsin2u⋅∑n=2∞(−iy−1sinu)nxn−1Jn(2πλ−1ysinu))exp(iβ).

**Figure 2:**
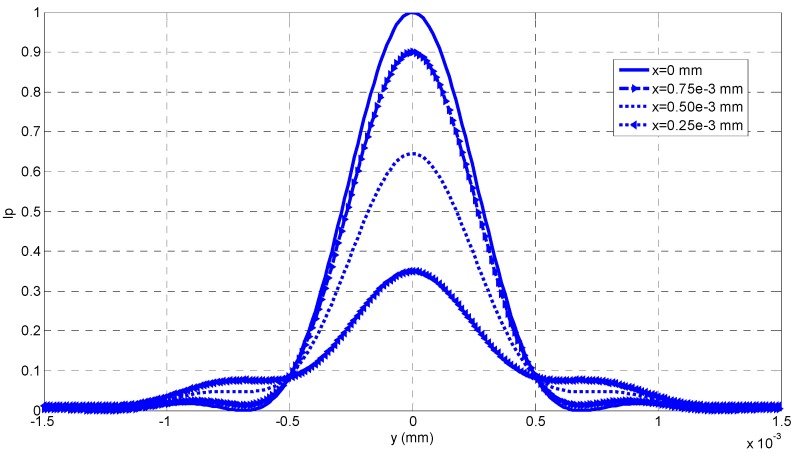
The intensity distribution curves of a random point *P*.

From Equations (9) and (10), we can see that the intensity distribution of a point on the imaging plane varies with *x* and *y*. In order to analyze the pattern of the intensity distribution, we fix the other parameters, such as *λ* = 600 nm, sin*u* = 0.5, and the intensity distribution with different *x* can be calculated, which is shown in Figure 2. From Figure 2, it can be seen that the intensity of *P* is maximal when the random point *P* coincides with the original point *O*, and the maximal value of the intensity decreases when *x* increases. On both sides of the original point *O*, the intensity value decreases with the increase of its distance from *O*. Furthermore, even though *x* = 0, *I*_p_(*x*, *y*) distributes as a blurred round light spot, rather than a point as expected. That means that even when the ideal focus-imaging-condition of the geometrical optics is satisfied, there is still an imaging blurring process.

**Figure 3:**
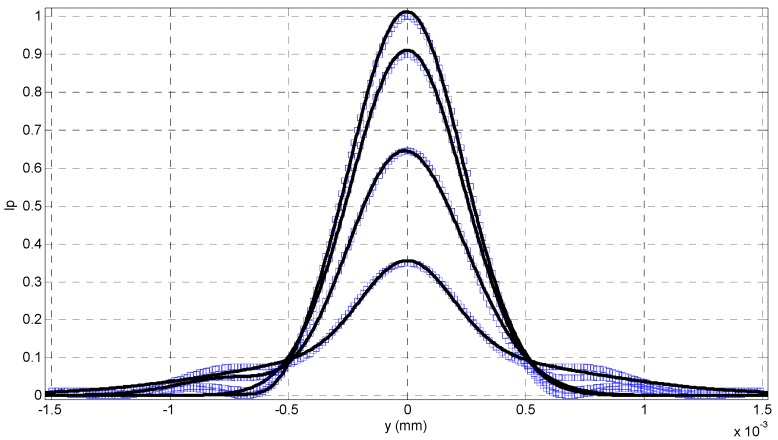
The fitted Gaussian curves with different depth variations.

From Figure 2, we can see that the intensity distribution *I*_p_(*x*, *y*) looks like a Gaussian function of *y* when *x* is fixed. Therefore, it is reasonable to fit each *I*_p_(*x*, *y*) with a Gaussian curve, and the fitted result can be seen in Figure 3, where the theoretical calculation from Equation (10) is denoted with the rectangular frames, and its fitted curves with a Gaussian function are drawn with the solid lines. Then, we can calculate the Gaussian kernel *σ* from each fitted Gaussian curve, and *σ* is called as blurring kernel which can evaluate the concentration of intensity distribution with respect to different *x*.

**Figure 4:**
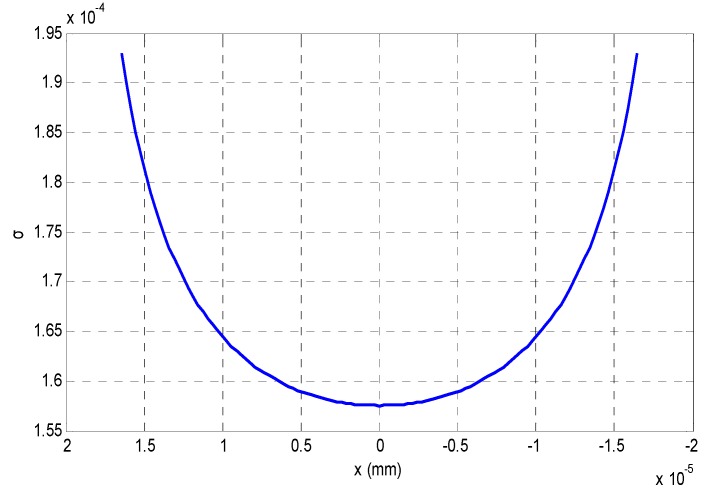
The burring degree of different depth variations.

Therefore, if we know the relationship between the blurring kernel and the depth variation from curve fitting, it is possible to reconstruct 3D shape of a scene from its blurring image, as shown in Figure 4. This relationship can be called as the blurring imaging model, which is constructed with the analysis of the intensity distribution with respect to variation of the object distance.

## 3. Depth Reconstruction with the Blurring Imaging Model

**Figure 5:**
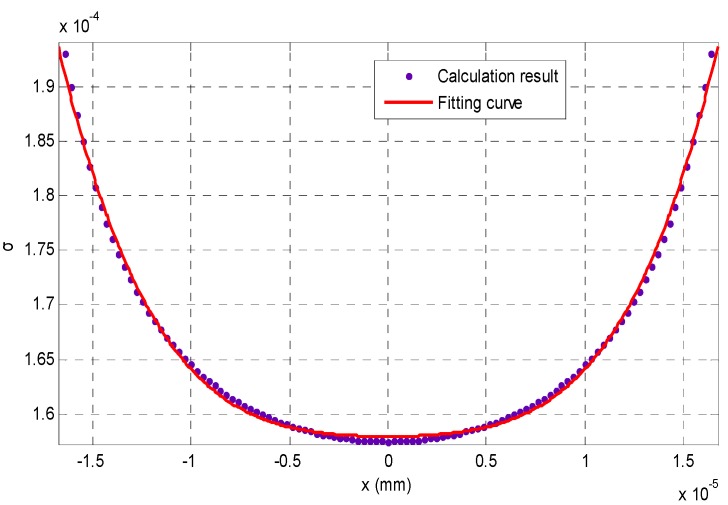
The blurring imaging model fitted by a 4th order polynomial curve.

From the analysis in Section 2, we could find that a numerical imaging model between *x* and *σ* is required if we want to calculate the depth information from the degree of blurring of some blurred images, and the relationship between *σ* and *x* in Figure 4 can be fitted with a 4th order polynomial curve after the Gaussian fitting. Figure 5 is the relationship between *σ* and *x*.

A 4th order polynomial curve is used to fit:

(11) $$\sigma =p_{1}x^{4}+p_{2}x^{3}+p_{3}x^{2}+p_{4}x+p_{5}$$

The solution process is described as follows: first, normalize the coefficients of the fitting curve in Equation (11):

(12) $$a=1,\;b=\frac{p_{2}}{p_{1}},\;c=\frac{p_{3}}{p_{1}},\;d=\frac{p_{4}}{p_{1}},\;e=\frac{\left(p_{5}-\sigma \right)}{p_{1}}$$

A cubic equation can be obtained to solve the 4th order equation in Equation (11):

(13) $${y_{t}}^{3}+q{y_{t}}^{2}+my_{t}+n=0$$

where q=−c, m=bd−4e, $n=-d^{2}-b^{2}e+4ce$.

The solution of Equation (13) is:

(14) $$y_{t}=\sqrt[{3}]{-\frac{\frac{2q^{3}}{27}-\frac{qm}{3}+n}{2}-z}+\sqrt[{3}]{-\frac{\frac{2q^{3}}{27}-\frac{qm}{3}+n}{2}+z}+\frac{c}{3}$$

where: $z=\sqrt{\left|\frac{{\left(\frac{2q^{3}}{27}-\frac{qm}{3}+n\right)}^{2}}{4}+{\left(\frac{-\frac{q^{2}}{3}+m}{3}\right)}^{3}\right|}$.

Finally, the depth variation can be denoted as:

(15) $$\left\{ \begin{array}{l} x=\frac{-(\frac{1}{2}b+\bar{s})\pm \sqrt{{\left(\frac{1}{2}b+\bar{s}\right)}^{2}-4\left(\frac{1}{2}y_{t}+\hat{s}\right)}}{2}\\ x=\frac{-(\frac{1}{2}b-\bar{s})\pm \sqrt{{\left(\frac{1}{2}b-\bar{s}\right)}^{2}-4\left(\frac{1}{2}y_{t}-\hat{s}\right)}}{2} \end{array}\right.$$

where: $\bar{s}=\sqrt{\left|\frac{1}{4}b^{2}-c+y_{t}\right|};\hat{s}=\sqrt{\left|\frac{1}{4}{y_{t}}^{2}-e\right|}$.

The finial depth is equal to:

(16) $$s=s_{0}+x$$

From Equation (11), we can find that after the curve fitting it is easy to know *p*_1_ to *p*_5_ in Equation (11). Therefore, there is only one unknown parameter, the blurring kernel *σ*, excepted for the desired depth information. In this paper, we will analyze the basic principle of blurring imaging, and then construct the formula of the blurring kernel *σ*.

When a real aperture camera is analyzed, the blurred image *E* captured on the imaging plane can be approximated with the following equation:

(17) E(y,z)=∬h(y,z,r)I(u,v)dudv

where *r* is the radius of the blurring round spot; *y* and *z* are the horizontal and the vertical coordinates of an image point, respectively; *h* is the point spread function (PSF) of the intensity distribution; *E*(*y*, *z*) is the blurring image; *I*(*y*, *z*) is the radiance image.

If the scene we consider is made of an Equifocal plane, which is parallel to the image plane, the depth map of the plane and the PSF *h*, which is shift-invariant, can be denoted as *s*(*y, z*) = *s* and *h*(*y*, *z*, *r*) *=* *h*(*y* − *z*, *r*), respectively. Therefore, the image formation model can be denoted by a simple convolution:

(18) E(y,z)=h(y,z)∗I(y,z)

where “*” denotes convolution in mathematics.

From Figure 2, we can find that for most of the depth variation the PSF can be denoted as a Gaussian function:

(19) $$h(y,z,y_{1},z_{1})=\frac{1}{2\pi \sigma^{2}}\exp(-\frac{{\left(y-y_{1}\right)}^{2}+{\left(z-z_{1}\right)}^{2}}{2\sigma^{2}})$$

where *σ* is the concentration parameter of a Gaussian function, and it is called as the Gaussian kernel. *σ* = *ζ r*, where *ζ* is a calibration parameter.

Then, the image model in Equation (17) can be formulated with the heat equation in Equation (5):

(20) $$\left\{ \begin{array}{l} \dot{u}(y,z,t)=\varepsilon \Delta u(y,z,t)\;\varepsilon \in [0,\infty )\;t\in (0,\infty )\\ u(y,z,0)=r_{0}(y,z) \end{array}\right.$$

where *r*_0_(*x*, *y*) is the radiance image without any blurring, the solution *u* at a time *t* is an image *I*(*x*, *y*) = *u*(*x*, *y*, *τ*), and the certain setting is related to the certain time *τ*. The variance *σ* is related to the diffusion coefficient *ε* with:

(21) $$\sigma^{2}=2t\varepsilon$$

When the PSF is shift varying, the equivalence with the isotropic heat equation cannot describe the complicated diffusion property, and the diffusion process can be formulated by the inhomogeneous diffusion equation in Equation (6):

(22) $$\left\{ \begin{array}{l} \dot{u}(y,z,t)=\nabla \cdot (\varepsilon (y,z)\nabla u(y,z,t)\;)\;t\in (0,\infty )\\ u(y,z,0)=r_{0}(y,z) \end{array}\right.$$

The relationship between the diffusion coefficient *ε* and the space-varying variance *σ* can be denoted as:

(23) $$\sigma^{2}(y,z)=2t\varepsilon (y,z)$$

Suppose there are two images *E*_1_(*y*, *z*) and *E*_2_(*y*, *z*) for two different blurring settings *σ*_1_ and *σ*_2_, and *σ*_1_ < *σ*_2_ (that is, *E*_1_(*y*, *z*) is less blurring than *E*_2_(*y*, *z*), (that is *σ*_1_ < *σ*_2_), from Equation (18), then *E*_2_ (*y*, *z*) can be denoted:

(24) $$\begin{array}{l} E_{2}\left(y,z\right)=\iint h(y,z,\sigma_{2}^{2})I(u,v)dudv\\ =\iint \frac{1}{2\pi \sigma_{2}^{2}}exp(-\frac{{\left(y-u\right)}^{2}+{\left(z-v\right)}^{2}}{2\sigma_{2}^{2}})I(u,v)dudv\\ =\iint \frac{1}{2\pi (\sigma_{2}^{2}-\sigma_{1}^{2})}\exp(-\frac{{\left(y-u\right)}^{2}+{\left(z-v\right)}^{2}}{2(\sigma_{2}^{2}-\sigma_{1}^{2})})dudv\cdot \iint \frac{1}{2\pi \sigma_{1}^{2}}\exp(-\frac{{\left(u-\tilde{y}\right)}^{2}+{\left(v-\tilde{z}\right)}^{2}}{2\sigma_{1}^{2}})I(\tilde{y},\tilde{z})d\tilde{y}d\tilde{z}\\ =\iint \frac{1}{2\pi \Delta \sigma^{2}}\exp(-\frac{{\left(y-u\right)}^{2}+{\left(z-v\right)}^{2}}{2\Delta \sigma^{2}})E_{1}(u,v)dudv\\ =\iint h(y,z,\Delta \sigma^{2})E_{1}(u,v)dudv\\ =h(y,z,\Delta \sigma^{2})*E_{1}(u,v) \end{array}$$

where $\Delta \sigma^{2}=\sigma_{2}^{2}-\sigma_{1}^{2}$, is the relative blurring between the blurred images *E*_1_(*y*, *z*) and *E*_2_(*y*, *z*). Therefore, a blurred image formulated by another blurred image taking into account relative blurring without the help of the radiance image.

Suppose *E*_1_(*y*, *z*) with the depth map of *s*_1_(*y*, *z*) is the first blurred image captured before a depth variation; *E*_2_ (*y*, *z*) with the depth *s*_2_(*y*, *z*) is another blurred image captured after depth variation; Δ*s*(*y*, *z*) is the depth variation along the optical axis; *s*_0_ is the ideal object distance. From the analysis above, we could find that if Δ*s* is known, it is reasonable to calculate the initial depth *s*_1_(*y*, *z*) from the heat diffusion equations.

First, the imaging process between two blurred images of the same scene can be denoted Equation (24), and based on it the blurred imaging process between *E*_1_(*y*, *z*) and *E*_1_(*y*, *z*) can be denoted with the heat diffusion equations:

(25) $$\left\{ \begin{array}{l} \dot{u}(y,z,t)=\nabla \cdot (\varepsilon (y,z)\nabla u(y,z,t)\;)\;t\in (0,\infty )\\ u(y,z,0)=E_{1}(y,z)\\ u(y,z,\Delta t)=E_{2}(y,z) \end{array}\right.$$

The relative blurring between them is:

(26) $$\Delta \sigma^{2}=\sigma_{2}^{2}-\sigma_{1}^{2}={\left(p_{1}{x_{2}}^{4}+p_{2}{x_{2}}^{3}+p_{3}{x_{2}}^{2}+p_{4}x_{2}+p_{5}\right)}^{2}-{\left(p_{1}{x_{1}}^{4}+p_{2}{x_{1}}^{3}+p_{3}{x_{1}}^{2}+p_{4}x_{1}+p_{5}\right)}^{2}$$

Then, the time Δ*t* can be taken as the variable which describes the global amount of blurring, and the diffusion coefficient *ε* can be denoted as:

(27) $$\varepsilon =\frac{\Delta \sigma^{2}}{2\Delta t}$$

Because our method is a global algorithm, an optimization problem is required to solve the diffusion equations globally, and the optimization problem can be transformed as:

(28) $$\tilde{s}=\underset{s_{2}(y,z)}{\arg\min}{\int \int \left(u(y,z,\Delta t)-E_{2}(y,z)\right)}^{2}dydz$$

However, the optimization process in Equation (28) is ill posed in mathematics, which means, it is difficult to find the minimum solution from Equation (28). Therefore, we regularize the problem with a Tikhonov Penalty:

(29) $$\tilde{s}=\underset{s_{2}(y,z)}{\arg\min}{\int \int \left(u(y,z,\Delta t)-E_{2}(y,z)\right)}^{2}dydz+\alpha {\left\|\nabla s_{2}(y,z)\right\|}^{2}+\alpha k{\left\|s_{2}(y,z)\right\|}^{2}$$

where the additional term imposes a smoothness constraint on the depth map. In practice, we can define that α>0, υ>0 and they are too small to introduce additional influence on the cost energy, denoted as:

(30) $$D(s)={\int \int \left(u(y,z,\Delta t)-E_{2}(y,z)\right)}^{2}dydz+\alpha {\left\|\nabla s\right\|}^{2}+\alpha \upsilon {\left\|s\right\|}^{2}$$

Thus the solution process is transformed into:

(31) $$\tilde{s}=\arg\underset{s}{\min}D(s)\;$$

**Figure 6:**
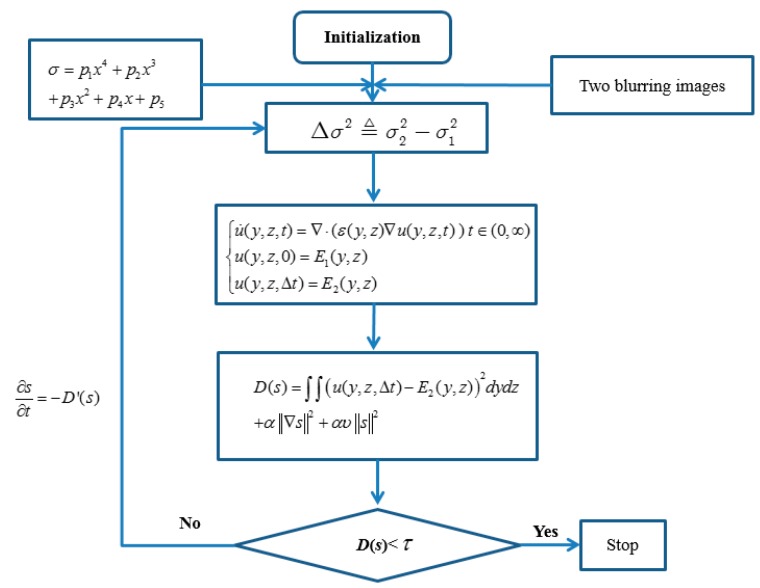
The process diagram of our method.

The flow-process diagram of our algorithm is shown as Figure 6. Our algorithm in this paper can be described by the following six steps:

(1) Give camera parameters *R*, *ζ*, and *s*_0_; two blurring images *E*_1_, *E*_2_; a threshold *τ*; *α*, *v*, and the step size *β* of each step;
(2) Initialize the depth map an equifocal plane;
(3) Calculate Equation (26) and obtain the relative blurring;
(4) Solve the diffusion equations in Equation (25) and attain the solution u(y,z,Δt);
(5) Based on u(y,z,Δt) calculate Equation (30). If the cost energy in Equation (30) is less thanε, the algorithm stops, and the finial depth map is obtained; otherwise calculate the following equation with step *β*:
  (32) $$\frac{\partial s}{\partial t}=-D'(s)$$
(6) Calculate Equation (27), update the global depth information, and return to Step 3.

## 4. Experiment

**Figure 7:**
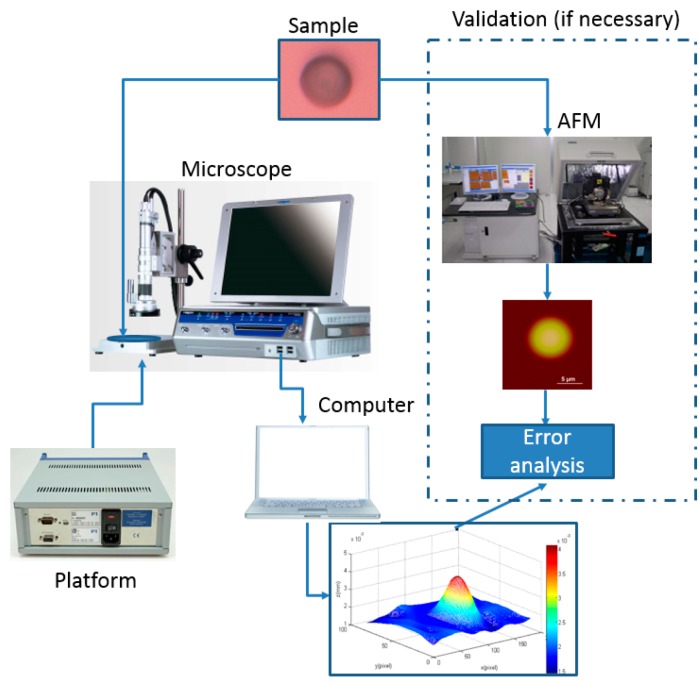
The connection of our experiment.

We validate our algorithm in this paper with a nanogrid, an AFM cantilever, and a microlens among a microlens array. The reason that we choose them as our samples is that their shape has been structured or controlled precisely at the micro/nanometer scale. In the first experiment, a nanogrid, where the step height is 500 nm and each pitch is 1 μm, is placed on a Physik Instrumente (PI) nanoplatform, and its shape is reconstructed with our algorithm. The connection of our experiment is shown as Figure 7.

In the second experiment, the PI nanoplatform is placed tightly up to the tip of the AFM cantilever, and the raised height of the platform is 100 nm. Then, we use our algorithm to reconstruct the shape when the AFM cantilever is bent to the expected height. In the third experiment, the geometrical shape of each microlens is a hemispheroid. Its average radius along the vertical direction is 2 μm, and its average radius along the horizontal direction is 6.5 μm. The depth variation Δs is controlled by the PI platform where the microlens is placed. In order to evaluate the reconstructed shape of our algorithm, we use a Veeco Dimension 3100 AFM system to scan the microlens before the shape reconstruction. The scanning frequency of the AFM system is 1 Hz. In these experiments, the microscope that we use is a HIROX-7700 with an amplification factor of 7000. The remaining parameters of the microscope are a focal length f = 0.357 mm, s_0_ = 3.4 mm, and Δs = 100 nm.

### 4.1. Nano Grid

**Figure 8:**
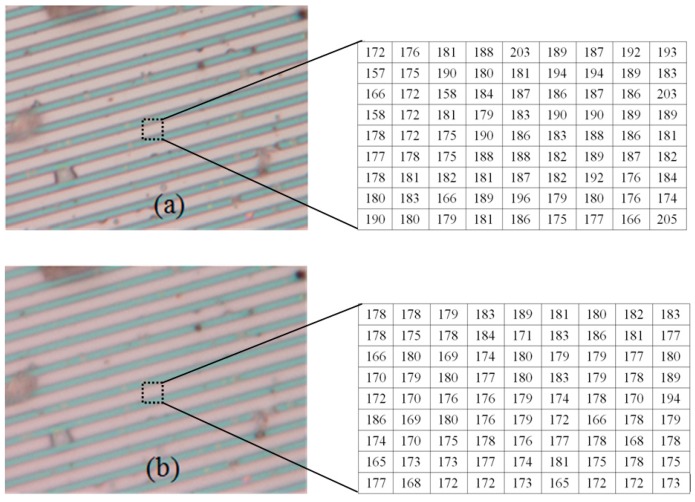
The blurred images of a nanogrid (the intensity values in the dotted rectangles are presented with two matrixes). (**a**) The blurred image before raising the platform; (**b**) the blurred image after raising the platform.

**Figure 9:**
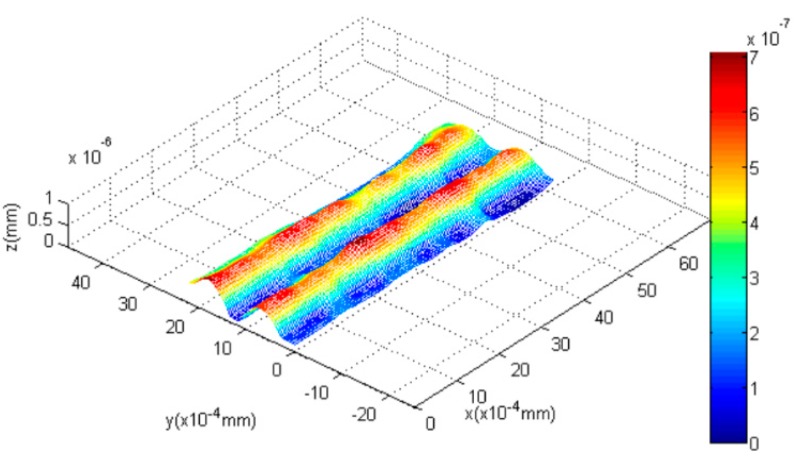
The reconstructed shape of the grid.

**Figure 10:**
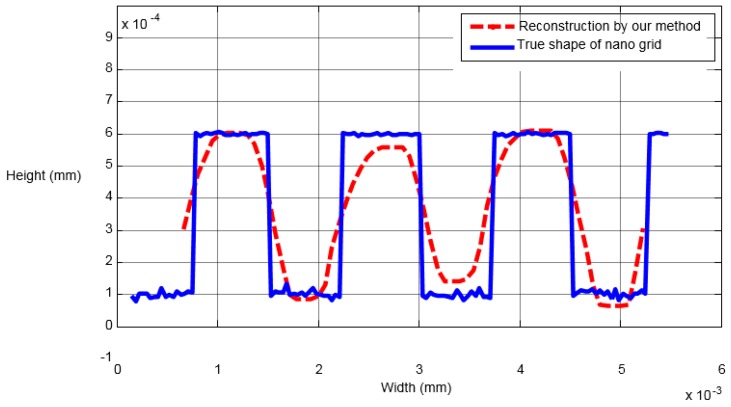
The comparison of an arbitrary section.

First, we capture the first blurred image of the nanogrid before rising the PI nanoplatform, and then the PI nanoplatform is raised by 100 nm, we capture another blurred image of the grid. Finally, we reconstruct the 3D shape of the nanogrid with these two blurred images, and the results can be seen in Figure 8, Figure 9 and Figure 10. Figure 8a is the blurred image before the PI platform is raised and Figure 8b is that after rising, and the intensity values in the area of the dotted rectangle are presented with a matrix; the reconstructed shape is shown in Figure 9. Figure 10 is a section comparison between the reconstructed shape and the true shape of the nanogrid. The unit of the depth axis in Figure 9 is mm.

From Figure 10, we can find that our reconstruction method can reconstruct the shape variation precisely compared to the true ground, and the maximal height error of the reconstructed shape appears at the top of the second pitch, which is 0.03 μm.

### 4.2. AFM Cantilever

**Figure 11:**
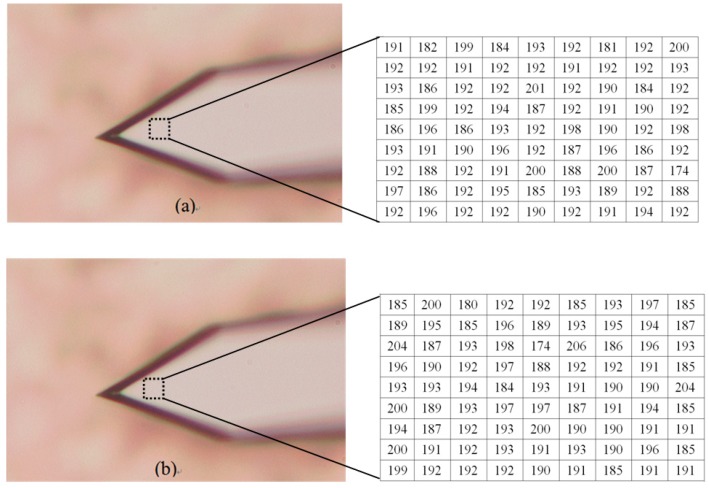
The blurred images of the tested AFM cantilever (the intensity values in the dotted rectangles are presented with two matrixes). (**a**) The blurred image before the platform is raised; (**b**) The blurred image after raising the platform.

**Figure 12:**
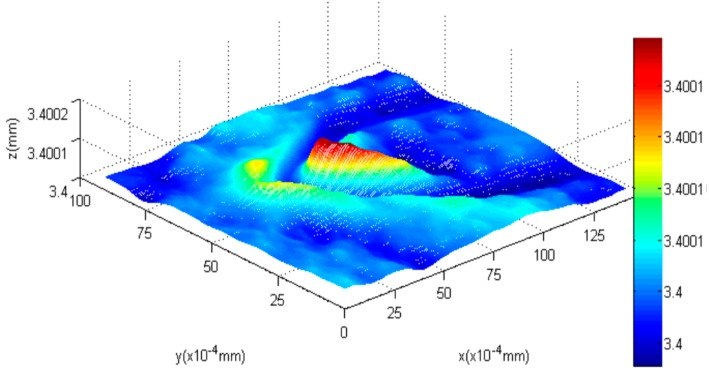
The reconstructed 3D shape of the cantilever.

**Figure 13:**
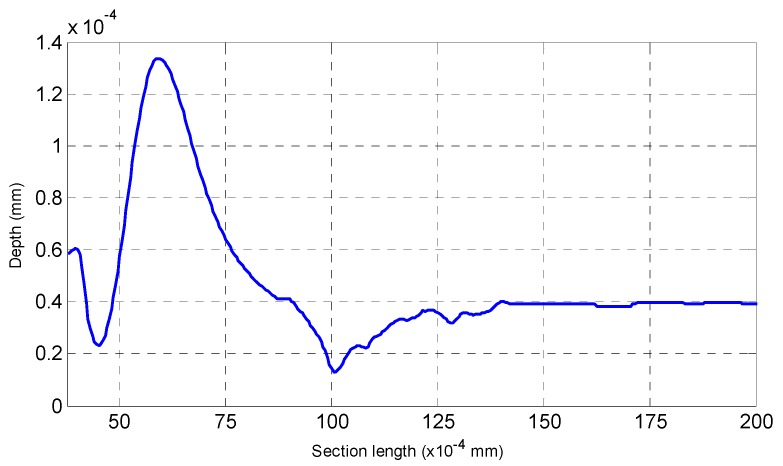
An arbitrary section of the reconstructed AFM cantilever.

Second, the AFM cantilever is tested. First, the first blurred image of the AFM cantilever before the PI platform is raised is obtained. After the PI nanoplatform rises by 100 nm, we capture another blurred image of the AFM cantilever. Then, the shape of the bent cantilever is reconstructed with our algorithm, and the results can be seen in Figure 11, Figure 12 and Figure 13. Figure 11a is the blurred image before the PI platform rising and Figure 11b is the blurred image after rising; Figure 12 is the reconstructed shape of the AFM cantilever. Figure 13 is a random profile of the bended AFM cantilever. In Figure 13, in order to show the depth map clearly, a distance of 3.4 mm is subtracted from the vertical axis. The unit of the depth axis in Figure 13 is mm.

From Figure 12 and Figure 13, the following conclusion is obtained,

(1) The highest bend on the AFM cantilever is at the cantilever’s end with the tip, and the bend height between the highest point and lowest point is 97 nm (1.36 × 10^−4^ mm − 0.39 × 10^−4^ mm = 0.97 × 10^−4^ mm) using our method. Therefore, the error of the highest bent point is 3 nm.
(2) For the reconstructed shape of the platform, it is plane compared to the bent cantilever, which coincides with the experimental fact.
(3) On both sides of the bent tip, we can see distinct troughs next to the spike. The reason for this result is that in this paper a global optimization method, which requires a secular change until an optimization value is obtained, is used to solve the diffusion equations. Therefore, one of our future tasks will be to modify the solution method during the shape reconstruction process to solve this problem, and the research direction is to design some adaptive scheme to find an optimal threshold and an interactive step for different surfaces.

### 4.3. Microlens

**Figure 14:**
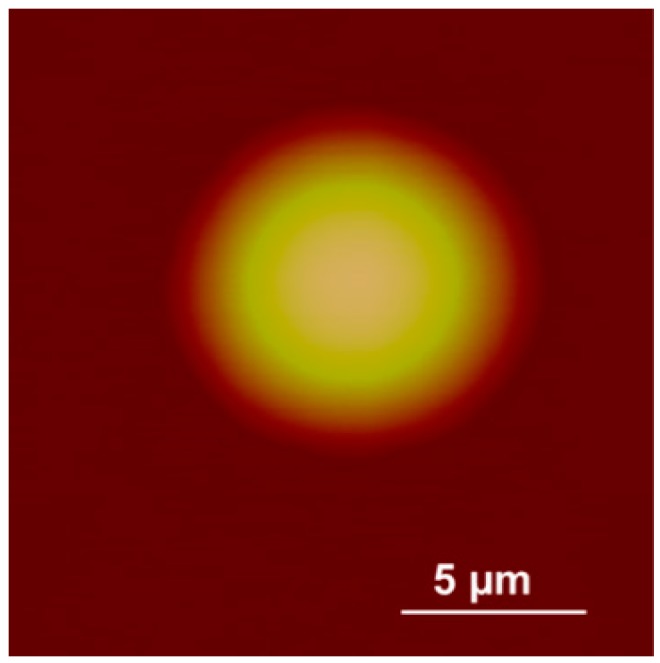
The image of the microlens scanned by AFM.

**Figure 15:**
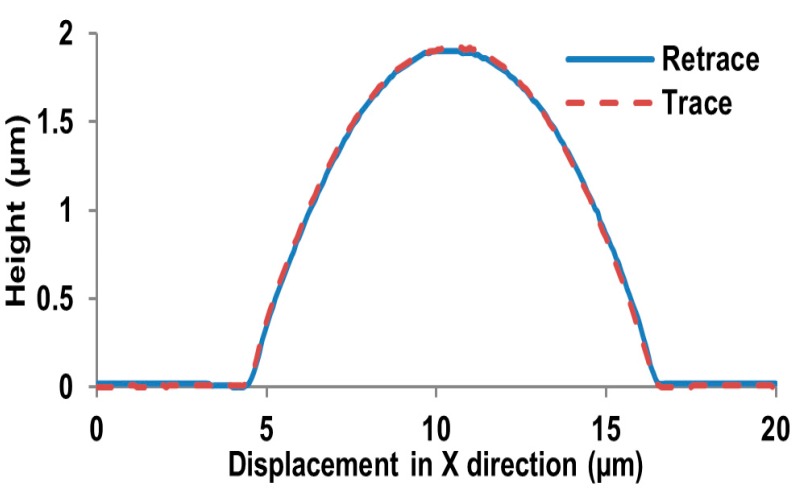
The profile of a section scanned by AFM.

**Figure 16:**
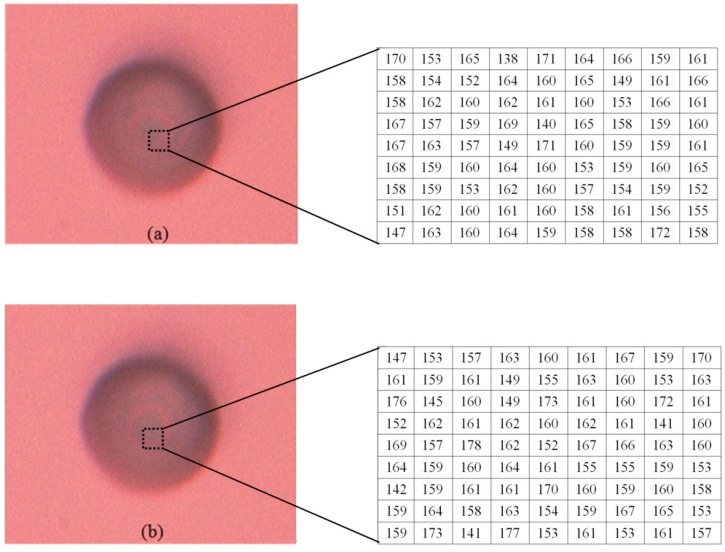
The blurred images of the microlens (the intensity values in the dotted rectangles are presented with two matrixes). (**a**) The blurred image before the platform is raised; (**b**) The blurred image after rising the platform.

**Figure 17:**
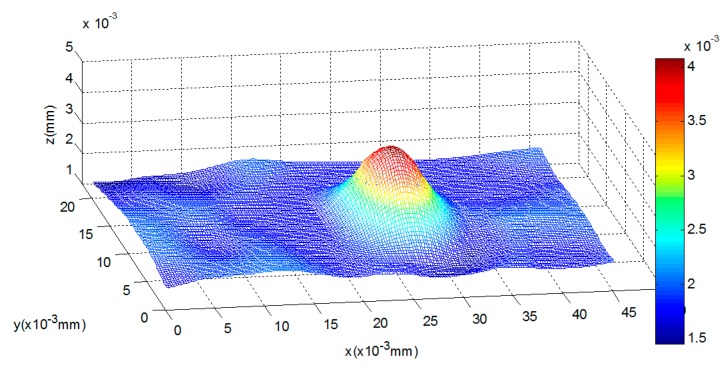
The reconstructed shape of the microlens with our method.

**Figure 18:**
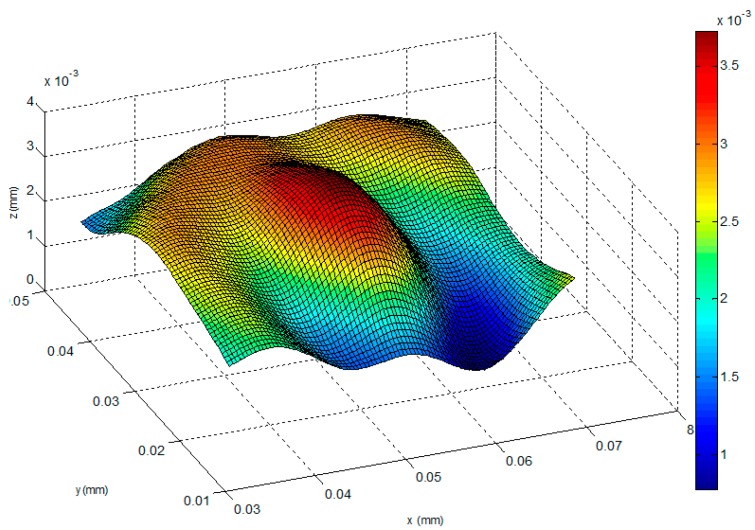
The reconstructed shape of the traditional SFD [6].

**Figure 19:**
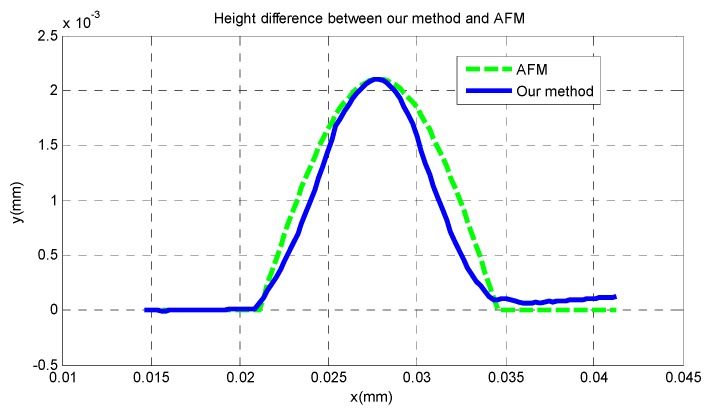
The profiles of the microlens with AFM and our method.

In this experiment, we capture the first blurred image of the microlens with our microscope, and control the PI platform to rise by 100 nm. Then, we capture the second blurred image and reconstruct the shape of the tested microlens with our algorithm and the traditional SFD without optical diffraction presented in reference [6], respectively. The experimental results are shown in Figure 14, Figure 15, Figure 16, Figure 17, Figure 18 and Figure 19. Figure 14 is 3D image of the microlens after AFM scanning and Figure 15, which shows that the horizontal radius of the microlens is 6.5 μm and its height is 2 μm, is the profile of any section from AFM scanning. Figure 16a is the blurred image before the platform is raised and Figure 16b is the blurred image after rising it; the reconstructed 3D shapes shown in Figure 17 and Figure 18 are the results of our new SFD and the traditional SFD, respectively.

From Figure 17, it can be seen that the entire shape of the microlens and the planar substrate are reconstructed at the same time. The radius of the hemisphere along the vertical direction is approximately 2 μm, and its horizontal radius is approximately 6.5 μm. In Figure 18 it is difficult to detect the boundary of the microlens from the reconstructed shape of the traditional SFD, and the planar substrate is not reconstructed as expected. Moreover, in order to compare the reconstruction result with AFM scanning, we cut a section of our reconstructed shape randomly and compare it with the result of AFM scanning, which is shown in Figure 19, where we can see that our reconstructed profile is close to the scanning profile of the AFM.

Table 1 is a comparison between AFM scanning, our 3D reconstruction method and the traditional SFD. From Table 1, we can find that compared to AFM scanning, the vertical error of our method is smaller than the horizontal error; the minimum error at the highest point is 3 nm, and the average error of our new algorithm is 53 nm, while the complete running time of our method is only 11% of the AFM scanning time. While for the traditional SFD, the vertical error and the horizontal error are both higher than those of our method in this paper; the minimum error at the highest point is 20 nm; the total working time is close to that of our method.

## 5. Conclusions

In this paper, a shape reconstruction method with an optical blurred imaging model at the micro/nanometer scale is proposed and tested with a standard grid, an AFM cantilever and a microlens. Our primary contribution here is to modify the blurred imaging model of Fresnel diffraction in optics, and to develop a direct relationship between intensity distribution of a source point and its depth information. Our second contribution is that a new blurred imaging model is proposed based on the curve fitting of a 4th order polynomial curve. In this way, the blurred imaging is combined with the heat diffusion equations which are solved by a dynamic optimization method. Finally, a standard nanogrid, an AFM cantilever and a microlens are tested with our proposed method at the micro/nanometer scale, respectively. The results prove that the proposed algorithm can reconstruct 3D shape with two blurred images, and the minimal reconstruction error is 3 nm.

## Figures and Tables

**Table 1:** Performance of our 3D reconstruction method.

Parameter	AFM	Our Method	Traditional SFD
Vertical measurement (mm)	2.10 × 10^−3^	2.103 × 10^−3^	1.98 × 10^−3^
Horizontal measurement (mm)	13.5 × 10^−3^	13.7 × 10^−3^	14.6 × 10^−3^
Running time (s, 256 × 256 pixels)	240	27	25

## References

[1] Overbaugh J., Bangham C.R.M. (2001). Selection forces and constraints on retroviral sequence variation. Science.

[2] Huang C., Wu L.Y. (2012). State technological development: A case of China’s nanotechnology development. World Dev..

[3] Guthold M., Falvo M.R., Matthews W.G., Paulson S., Washburn S., Erie D.A. (2000). Controlled manipulation of molecular samples with the nanomanipulator. IEEE ASME Trans. Mechatron..

[4] Rubio-Sierra F.J., Burghardt S., Kempe A. Atomotic force microscope based nanomanipulator for mechanical and optical lithography. Proceedings of the IEEE Conference on Nanotechnology.

[5] Wu L.D. (1993). Computer Vision.

[6] Wei Y.J., Dong Z.L., Wu C.D. (2012). Depth measurement using single camera with fixed camera parameters. IET Comput. Vis..

[7] Venema L.C., Meunier V., Lambin P., Dekker C. (2000). Atomic structure of carbon nanotubes from scanning tunneling microscopy. Phys. Rev. B.

[8] Tian X.J., Wang Y.C., Xi N., Dong Z.L., Tong Z.H. (2008). Ordered arrays of liquid-deposited SWCNT and AFM manipulation. Sci. China.

[9] Yin C.Y. (1999). Determining residual nonlinearity of a high-precision heterodyne interferometer. Opt. Eng..

[10] Girod B., Scherock S. Depth from defocus of structured light. Proceedings of the Optics, Illumination, and Image Sensing for Machine Vision.

[11] Favaro P., Burger M., Osher S.J. (2008). Shape from defocus via diffusion. IEEE Trans. Pattern Anal. Mach. Intell..

[12] Favaro P., Mennucci A., Soatto S. (2003). Observing shape from defocused images. Int. J. Comput. Vis..

[13] Navar S.K., Watanabe M., Noguchi M. (1996). Real-time focus range sensor. IEEE Trans. Pattern Anal. Mach. Intell..

[14] Word R.C., Fitzgerald J.P.S., Konenkamp R. (2013). Direct imaging of optical diffraction in photoemission electron microscopy. Appl. Phys. Lett..

[15] Kantor I., Prakapenka V., Kantor A., Dera P., Kurnosov A., Sinogeikin S., Dubrovinskia A., Dubrovinsky L. (2012). BX90: A new diamond anvil cell design for X-ray diffraction and optical measurements. Rev. Sci. Instrum..

[16] Oberst H., Kouznetsov D., Shimizu K., Fujita J., Shimizu F. (2005). Fresnel diffraction mirror for atomic wave. Phys. Rev. Lett..

[17] Wang Z.Q., Wang P. (2000). Rayleigh criterion and K Strehl criterion. Acta Photonica Sin..

